# Update and optimization of a multiplex RT-qPCR assay to overcome diagnostic failure in emerging influenza A(H3N2) subclades J.2 and K (Peru, 2024–2026)

**DOI:** 10.1016/j.ijregi.2026.100993

**Published:** 2026-09-01

**Authors:** Miguel Angel Aguilar-Luis, Juana del Valle-Mendoza, Yordi Tarazona-Castro, Deysi Aguilar-Luis, Eliezer Bonifacio-Velez de Villa, Angela Cornejo-Tapia, Andrés Antón, Cristina Andrés, Alberto Laguna-Torres

**Affiliations:** a Biomedicine laboratory, Research and Innovation Centre of the Faculty of Health Sciences, Universidad Peruana de Ciencias Aplicadas, Lima, Peru; b Instituto de Investigación Nutricional, Lima, Peru; c Vall d'Hebron Institut de Recerca, Barcelona, Spain; d Centro de Investigacion Biomedica en Red Enfermedades Infecciosas, Madrid, Spain

**Keywords:** Influenza A(H3N2), Subclade K, Multiplex RT-qPCR, Genomic surveillance, Molecular diagnostics, Peru

## Abstract

**Objectives:**

Continuous genetic drift in the hemagglutinin gene of influenza A(H3N2) viruses compromises the reliability of standard molecular diagnostics, resulting in target dropouts and epidemiological surveillance gaps. This study evaluated the clinical performance of the World Health Organization (WHO)-recommended (2024) real-time reverse transcription-quantitative polymerase chain reaction (RT-qPCR) sets against emerging Peruvian A(H3N2) subclades J.2 and K, and developed an optimized dual-target multiplex assay to restore diagnostic sensitivity.

**Methods:**

Genomic sequencing was used to identify mutational profiles in circulating Peruvian A(H3N2) subclades J.2 and K. Standard WHO (2024) oligonucleotides were tested against 20 A(H3N2)-confirmed clinical samples. The dual-target multiplex RT-qPCR assay was then redesigned with targeted bioinformatic modifications and validated.

**Results:**

Genomic analysis identified critical oligonucleotide mismatches in both subclades. Consequently, the standard WHO assay failed to detect the virus in any tested isolate. The optimized multiplex assay successfully restored amplification in all 20 sequencing-confirmed A(H3N2) viruses. It demonstrated high analytical concordance (Pearson’s r = 0.70, *P* < 0.001) between the two targets without competitive inhibition. Furthermore, both the J.2 and the highly mutated K subclades were amplified with equivalent diagnostic performance.

**Conclusion:**

The optimized dual-target RT-qPCR assay successfully overcomes the diagnostic failure of standard probes, providing a reliable, fail-safe mechanism for detecting currently circulating H3N2 variants. These findings emphasize the necessity of routine genomic audits to maintain current molecular protocols designed for detection.

## Highlights

• Standard World Health Organization reverse transcription-quantitative polymerase chain reaction probes fail to detect emerging A(H3N2) J.2 and K subclades.
• Genomic audits identified critical mutations causing target dropouts in Peru.
• An optimized dual-target multiplex assay restores full diagnostic sensitivity.
• The redesign achieved 100% specificity without competitive inhibition.
• Routine genomic monitoring is essential to update frontline molecular assays.

## Introduction

Influenza A(H3N2) viruses represent a persistent and dynamic challenge to global public health, characterized by a substantially higher disease burden and a more rapid rate of molecular evolution than other seasonal respiratory viruses [1]. Effective epidemiological surveillance and clinical response are based on the precision of molecular diagnostics, including real-time reverse transcription-quantitative polymerase chain reaction (RT-qPCR), the gold standard for subtyping influenza viruses [2,3]. However, the long-term efficacy of these molecular tools is intrinsically linked to the sequence stability of target regions within the hemagglutinin (HA) gene, which continuously accumulate synonymous and non-synonymous mutations under constant immunological selective pressure [4,5].

Despite the existence of consolidated international protocols, such as those issued by the World Health Organization (WHO) in 2024 and the Centers for Disease Control and Prevention in 2021, genetically divergent variants have begun to erode the analytical sensitivity of traditional primer and probe sets [3,6]. Between 2024 and 2026, a critical epidemiological transition was observed, marked by the consolidation of 3C.2a1b.2a.2a.3a.1 and its subclades, specifically J.2 and K [7]. These subclades show highly modified amino acid profiles in key antigenic sites and receptor-binding domains, suggesting a possible mismatch with recommended vaccine strains [4,[8], [9], [10]] and compromising the hybridization kinetics of oligonucleotides used in routine diagnostic workflows.

The risk of molecular target failure caused by the natural genetic drift of the viral genome is well recognized in the field of ribonucleic acid (RNA) virus diagnostics. During the COVID-19 pandemic, point mutations in the severe acute respiratory syndrome coronavirus 2 S-gene caused ``target dropout'' events, which necessitated a reevaluation of PCR designs to prevent false negatives [11,12]. In the context of influenza A(H3N2), the risk is analogous: mismatches at the 3′ end of primers or in the central regions of TaqMan probes may result in significant delays in quantification cycles (Cq) or the loss of fluorescent signal [9,10]. Extended influenza seasons increase the impact of underreporting, complicating surveillance efforts within a One Health environment [7,13].

In South America, and specifically in Peru, influenza A(H3N2) circulation dynamics have shown patterns of local spread and persistence that demand robust genomic monitoring [14,15]. The integration of third-generation sequencing technologies, such as Oxford Nanopore Technologies (ONT), has allowed for unprecedented resolution in whole-genome characterization, enabling the real-time identification of mutations affecting molecular assay integrity [[16], [17], [18]]. However, the availability of high-throughput sequencing remains limited in many frontline clinical settings, and RT-qPCR is still the primary method for routine diagnosis in many settings [19,20]. Therefore, updating existing assays to reflect contemporary viral diversity is crucial to maintaining diagnostic sensitivity in frontline laboratories.

The present study addresses this technical gap through an evaluation of the performance of the standard WHO (2024) protocol using clinical samples collected in Peru between 2024 and 2026 and previously characterized by genomic sequencing. Following the confirmation of analytical sensitivity loss due to specific mutations in the J.2 and K lineages, we designed, optimized, and validated an updated multiplex RT-qPCR assay intended to restore reliable detection. This work not only provides an immediate solution to restore diagnostic precision against converging seasonal influenza threats [21] but also provides a practical framework for the dynamic revision of influenza molecular protocols in response to continuous viral evolution, thereby ensuring that early warning systems remain reliable [18].

## Materials and methods

### Sample collection

Clinical specimens, consisting of nasopharyngeal swabs, were obtained from hospitalized patients presenting with acute respiratory infection between July 2024 and February 2026. To preserve viral viability and nucleic acid stability, swabs were immediately inoculated into 3-mL tubes containing Universal Transport Medium-Room Temperature (RT) (Cat. # 330C, Copan Italia, Brescia, Italy). All samples were transported under strict cold chain conditions (4°C) and then stored at −80°C until molecular analysis was performed.

### Viral RNA extraction

Total viral RNA was extracted from 200 µL of Universal Transport Medium-preserved specimens using the High Pure Viral RNA Kit (Roche Diagnostics GmbH, Mannheim, Germany) following the manufacturer’s instructions. The extraction process utilized chemical lysis with guanidine hydrochloride and Proteinase K, followed by the selective binding of nucleic acids to a silica gel membrane in a spin-column format. After two sequential wash steps to remove PCR inhibitors and protein contaminants, purified RNA was eluted in 50 µL of nuclease-free buffer and immediately used for downstream applications or stored at −80°C.

### Genomic characterization

Initial influenza detection was performed as part of a routine real-time PCR diagnostic workflow. Positive influenza A specimens with a Cq value < 30 were prioritized for whole-genome sequencing (WGS). During the study period, we sequenced 84 influenza A-positive specimens, identifying a total of 28 influenza A(H3N2) cases (Supplementary Table S1). A subset of 20 WGS-confirmed specimens was selected to ensure a proportional representation of circulating variations. The 20 isolates were evenly distributed between two emerging subclades: 10 cases were classified as subclade J.2, and 10 as subclade K (Supplementary Table S2). This selected subset was included in the subsequent analysis.

Library preparation followed an amplicon-based approach in accordance with ONT (Oxford, UK) guidelines. Target enrichment for influenza A was performed using a universal primer scheme (Tuni 12: 5′-ACG CGT GAT CAG CAA AAG CAG G-3′; Tuni 12.4: 5′-ACG CGT GAT CAG CGA AAG CAG G-3′; Tuni 13: 5′-ACG CGT GAT CAG TAG AAA CAA GG-3′) synthesized by Macrogen (Seoul, South Korea). The one-step RT-PCR cycling parameters included reverse transcription at 42°C for 60 min and initial denaturation at 94°C for 2 min; followed by 5 cycles of 94°C for 30 s, 45°C for 30 s, and 68°C for 3 min; and a subsequent 31 cycles of 94°C for 30 s, 57°C for 30 s, and 68°C for 3 min. Sequencing libraries were constructed utilizing the Native Barcoding Kit 96 V14 (SQK-NBD114.96, ONT, Oxford, UK), which encompassed end-repair and dA-tailing (NEBNext Ultra II, New England Biolabs, MA, USA), native barcode ligation, and sequencing adapter attachment. Multiplexed libraries were loaded onto R10.4.1 Flow Cells and sequenced on a MinION device. Post-sequencing bioinformatic analysis, including quality filtering, alignment, and consensus sequence generation, was executed using the EPI2ME Desktop application (version 26.04-01) with the Influenza Typing Workflow (wf-flu version v1.3.1).

### RT-qPCR assay design and optimization

The RT-qPCR assay was optimized to target conserved regions of the HA gene in the emerging A(H3N2) J.2 and K subclades. The oligonucleotide sequences, including specific modifications to overcome previous hybridization failures, are listed in Table 1. Oligonucleotides were synthesized by Macrogen (Seoul, South Korea). Amplification was performed using the Luna® Universal Probe One-Step RT-qPCR Kit (New England Biolabs, Cat. # E3006). Each 20 µL reaction mixture consisted of 10 µL of Luna Universal Probe One-Step Reaction Mix (2 ×), 1 µL of Luna WarmStart® RT Enzyme Mix (20 ×), 0.8 µL of each primer (0.4 µM final concentration), 0.4 µL of the hydrolysis probe (0.2 µM final concentration), and 5 µL of template RNA.

**Table 1:** Oligonucleotide sequences for the optimized multiplex RT-qPCR assay targeting emerging influenza A(H3N2).

Target gene	Oligonucleotide designation	Sequence (5′→3′)	Fluorophore/quencher	Modifications/notes
HA (Set 1)	H3-266-F	ACCCTCAGTGTGATGGCTTTCAAA	—	WHO 2024Standard
	H3-266-F-Mod	GACCCTCAGTGTGATGGCTTTCA	—	5′ G addition, 3′ AA deletion
	H3-373-R	TAAGGGAGGCATAATCCGGCACAT	—	WHO 2024 Standard
	H3-315-Probe	ACGAAGCAAAGCCTACAGCAACTGTT	FAM / BHQ1	WHO 2024 Standard
	H3-315-Probe-Mod	ACGAAGCAGAGCCAACAGCAGCTGTT	ROX/BHQ-2	Three specific substitutions (A→G, T→A, A→G) to match J.2 and K subclades
HA (Set 2)	H3-666-F	GCACAGGGAATCTAATTGCTCC	—	WHO 2024 Standard
	H3-911-R	ATGCTTCCATTTGGAGTGATGCATTC	—	WHO 2024 Standard
	H3-732-Probe	GATCAGATGCACCCATTGGCAAATGC	FAM/BHQ1	WHO 2024 Standard
	H3-732-Probe-Mod	GATCAGACGCACCCATTGGCGAATGT	HEX/BHQ-1	Three specific substitutions (T→C, A→G, C→T), updated for J.2 and K subclades

Oligonucleotide modifications denote bioinformatic adjustments made to the original WHO 2024 standard sequences to restore thermodynamic stability and prevent target dropout against the highly mutated HA regions of contemporary subclades J.2 and K. Quenchers (BHQ-1/BHQ-2) correspond to the specific emission spectra of the selected fluorophores utilized in the Bio-Rad CFX Opus 96 system.

HA, hemagglutinin; WHO, World Health Organization.

The following conditions were used to conduct thermal cycling on a Bio-Rad CFX Opus 96 Real-Time PCR System: reverse transcription at 55°C for 10 min and initial denaturation at 95°C for 1 min, followed by 40 cycles of denaturation at 95°C for 10 s and combined annealing/extension at 60°C for 30 s. Fluorescent signals were acquired in the FAM, HEX, and ROX channels during the extension phase.

### Performance and validation

The performance evaluation was restricted to the clinical sensitivity and analytical specificity for influenza A(H3N2). The initial diagnostic workflow utilized previously validated screening primers targeting highly conserved genomic regions of the influenza virus, which proved highly effective for primary detection throughout the study period.

To evaluate the clinical impact of the observed mutations and establish a baseline for diagnostic failure, the previously characterized panel of 20 WGS-confirmed A(H3N2) isolates (n = 10 for subclade J.2; n = 10 for subclade K) was initially tested using the standard WHO-recommended (2024) oligonucleotides (Table 1). After this, a panel of 20 clinical samples for influenza A(H1N1)pdm09 and 20 influenza B were tested to determine analytical specificity and rule out cross-reactivity.

Due to the nature of the clinical specimens and the lack of quantified synthetic RNA, sensitivity was assessed by comparing the detection rate and distribution of Cq values between the standard WHO (2024) protocol and the newly optimized multiplex assay. This approach documented the restoration of diagnostic signals in samples with previously identified genomic mismatches.

### Statistical analysis and visualization

Cq values were summarized using descriptive statistics and reported as means with SDs. Diagnostic performance was assessed by evaluating clinical sensitivity (detection rate) against the characterized A(H3N2) clinical isolates and analytical specificity against the exclusion panel (A(H1N1)pdm09 and influenza B). As the standard WHO (2024)assay failed to produce quantifiable Cq values for the evaluated isolates, formal Cq comparisons were limited exclusively to the optimized multiplex targets.

Using paired-sample Student’s *t*-tests, Cq values obtained with modified Set 1 (ROX) and modified Set 2 (HEX) were compared, since both targets were evaluated simultaneously in the same A(H3N2) specimens from subclades J.2 and K. The Pearson correlation coefficient (r) and simple linear regression were used to assess the concordance between the two optimized targets and potential evidence of competitive inhibition in the dual-target format. Statistical significance was defined as a *P*-value of <0.05 for all two-tailed tests.

Statistical computations and primary data visualizations were conducted using OriginPro 2025 (version 10.2.0.188; OriginLab Corporation, Northampton, MA, USA). The final assembly of figures and vector formatting was performed in Inkscape (version 1.4.2).

### In silico validation of optimized oligonucleotides

To validate the applicability of the redesigned oligonucleotides, we performed an *in silico* analysis. We retrieved 103 complete, high-coverage (> 50 ×) influenza A(H3N2) HA sequences from the GISAID EpiFlu™ database, which were collected between January 2025 and August 2026. There were 71 isolates representing subclade J.2 and its derivatives (n = 71), 31 isolates representing subclade K (n = 31), and one unassigned contemporary lineage originating from 12 countries across four continents. Nextclade was used to align the sequences, and the inclusivity of the primer and probe sets (H3-266 and H3-666) was verified by aligning the target sequences against the representative H3N2 genomes (Supplementary Figures S1 and S2).

### Ethical considerations

The Institutional Ethics Committee of the Universidad Nacional Mayor de San Marcos approved the collection of the initial clinical samples (CIEI-2022-17), and written informed consent was obtained from all participants. The present study utilizing the de-identified remnants of these specimens has been approved by the Nutritional Research Institute (437-2026/CIEI-IIN). The ethics committee waived the requirement for an additional informed consent for this secondary use, ensuring patient anonymity and data confidentiality throughout the analytical procedure.

## Results

### In silico identification of genomic mismatches and WHO probe failure

**Figure 1:**
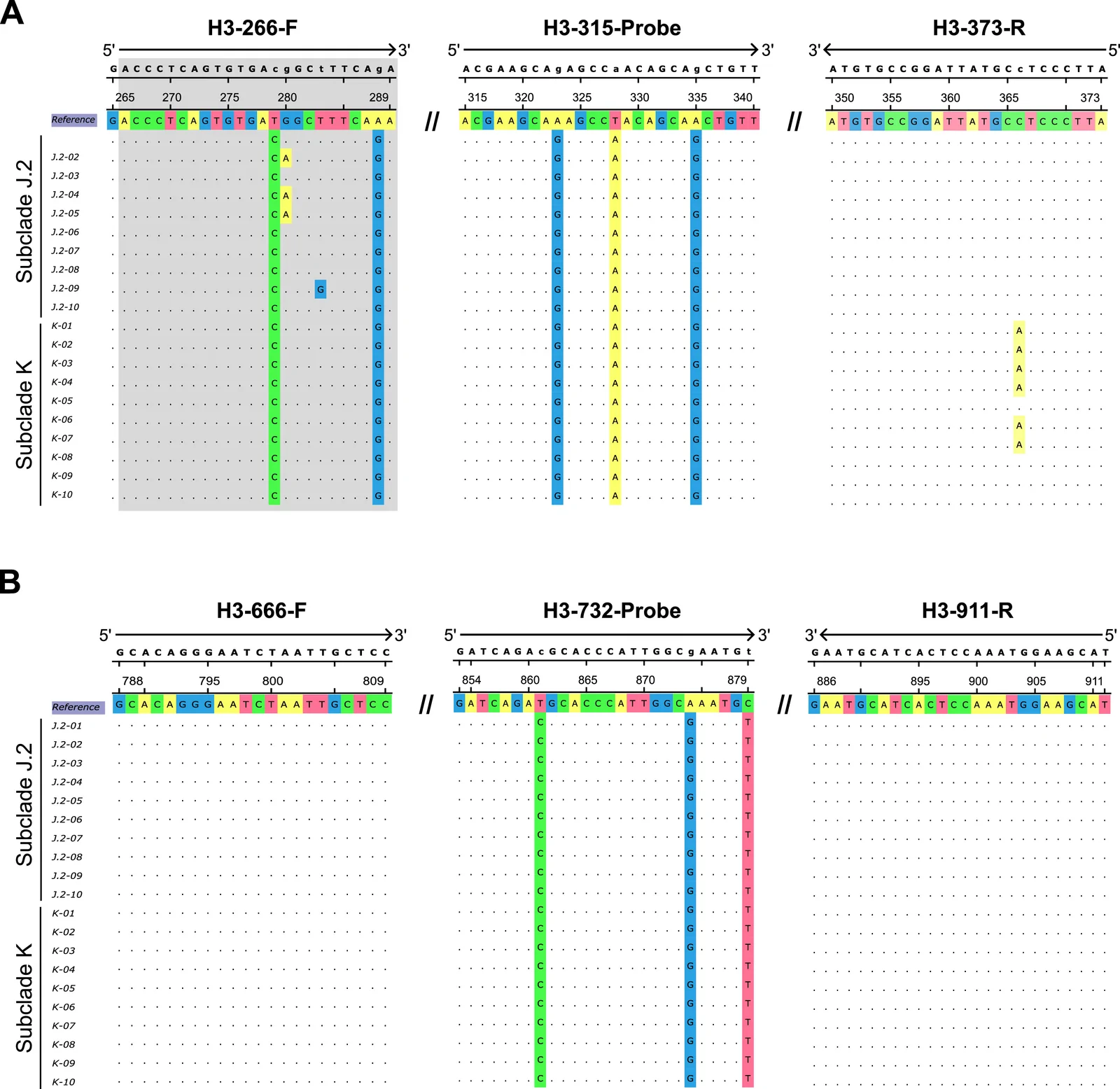
Sequence alignment of the influenza A(H3N2) HA gene in Peruvian isolates. (a) Genomic mismatches in the binding regions of Set 1 oligonucleotides (H3-266-F, H3-315-Probe, and H3-373-R). (b) Genomic mismatches in the binding regions of the Set 2 oligonucleotides (H3-666-F, H3-732-Probe, and H3-911-R). Highlighted nucleotides indicate the exact positions of these mismatches. The reference track represents an in silico consensus derived directly from the WHO 3standard oligonucleotide sequences. WHO, World Health Organization.

**Figure 2:**
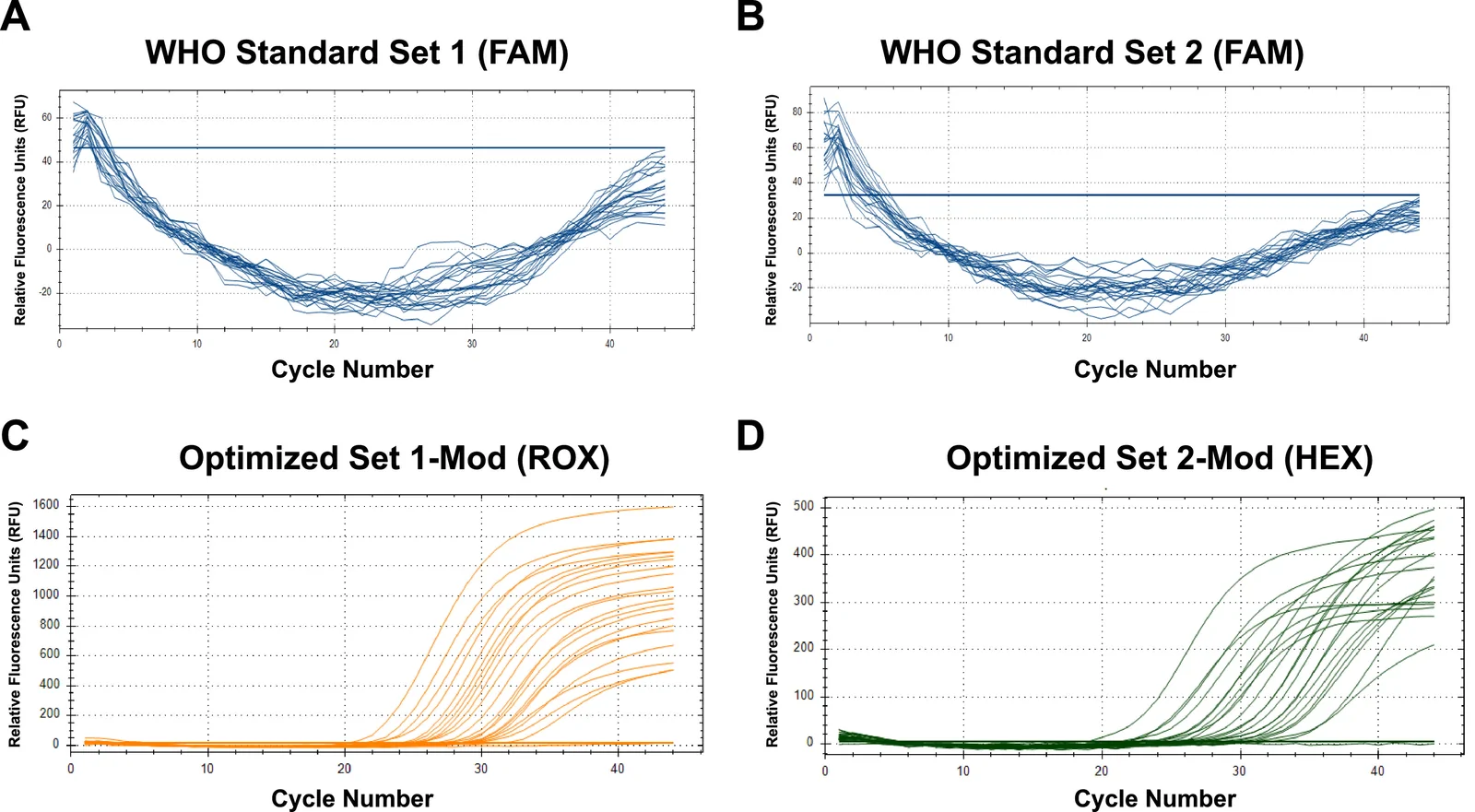
Real-time reverse transcription-quantitative PCR (RT-qPCR) amplification plots of influenza A(H3N2) diagnostic rescue. (a, b) Amplification curves using the original WHO standard oligonucleotide sequences. Both H3-315-Probe and H3-732-Probe targets (FAM-labeled) exhibited a complete failure to produce sigmoidal amplification curves across all tested J.2 and K subclade samples. (c, d) Restoration of diagnostic sensitivity using the optimized dual-target multiplex assay. The modified Set 1 (H3-315-Probe-Mod, ROX-labeled) and Set 2 (H3-732-Probe-Mod, HEX-labeled) generated robust, classical sigmoidal amplification profiles. Data were acquired using the Bio-Rad CFX Opus 96 system. WHO, World Health Organization.

Genomic analysis of the HA gene in circulating Peruvian influenza A(H3N2) isolates revealed significant genetic drift. Multiple sequence alignments revealed critical point mutations and mismatches within the contemporary J.2 and K subclades compared with the reference sequences used for standard diagnostic designs (Figure 1). Real-time PCR evaluation of these isolates using the standard WHO (2024)oligonucleotides confirmed complete diagnostic failure: neither the H3-315-Probe nor the H3-732-Probe targets (FAM-labeled) produced sigmoidal amplification curves, resulting in 0% sensitivity across all tested samples (Figure 2a and b).

### Dual-target multiplex assay optimization

To address the severe target dropout caused by the identified mutations, a modified dual-target multiplex assay was developed. Bioinformatic adjustments, including a 5′ G addition and a 3′ AA deletion for Set 1 and targeted sequence modifications for Set 2, were implemented to restore thermodynamic stability (Table 1). Amplification plots confirmed the successful rescue of diagnostic sensitivity. The optimized Set 1-Mod (ROX) and Set 2-Mod (HEX) generated robust, classical sigmoidal curves for all clinical isolates that had previously evaded detection (Figure 2c and d). Additionally, both optimized sets failed to exhibit cross-reactivity or nonspecific fluorescent signals when challenged with an exclusion panel containing 20 influenza A(H1N1)pdm09 and 20 influenza B specimens, confirming 100% analytical specificity for the targeted A(H3N2) lineages.

Furthermore, the optimized dual-target assay was validated *in silico* against 103 global A(H3N2) genome sequences. Sequence alignments demonstrated high conservation across the target regions for both the H3-266 and H3-666 sets, with no critical mismatches in the modified primer or probe binding sites (Supplementary Figures S1 and S2). The bioinformatic adjustments in Set 1-Mod and Set 2-Mod maintained exact complementarity with all evaluated international J.2 and K isolates.

### Analytical concordance

**Figure 3:**
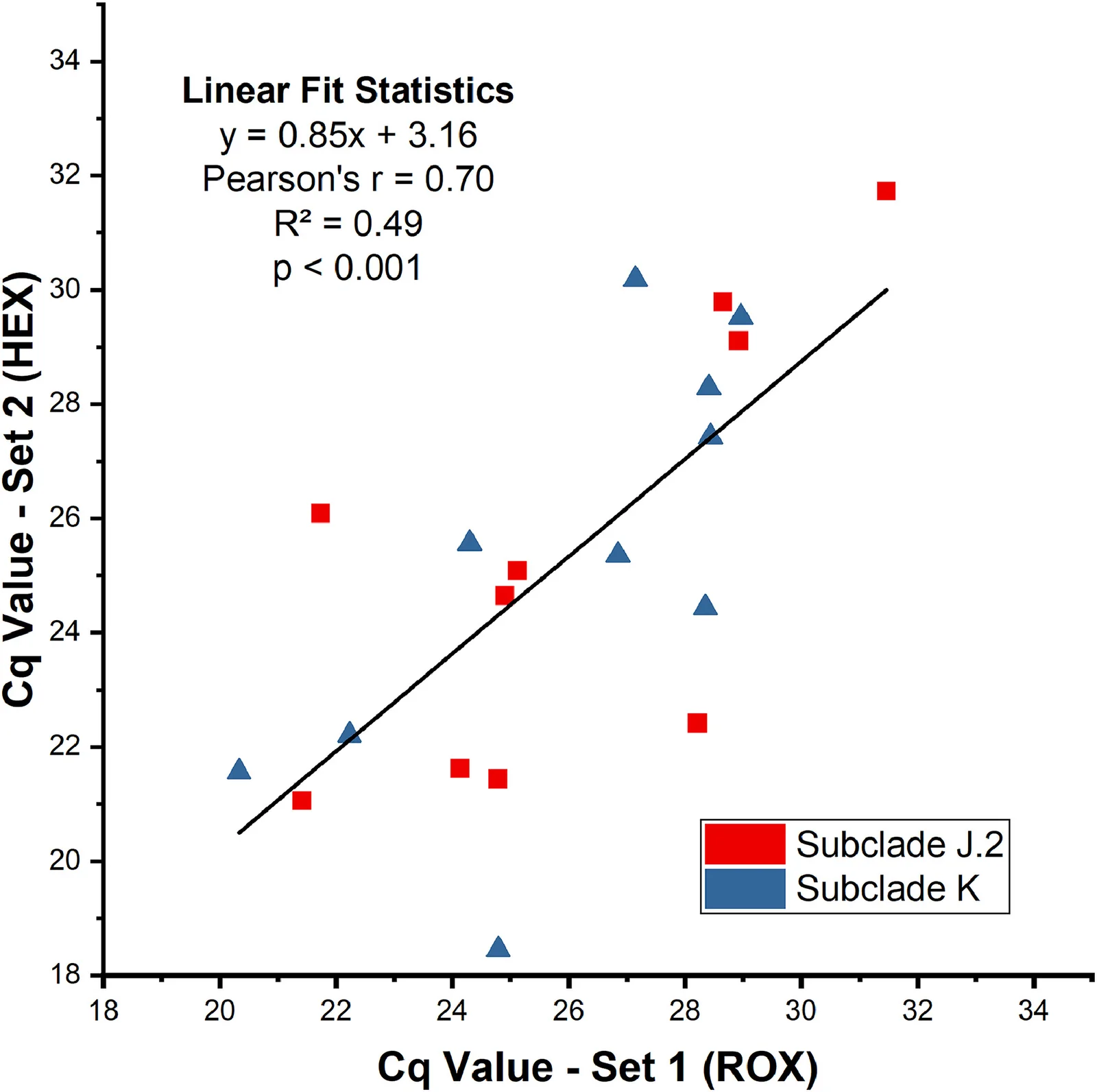
Analytical concordance of the optimized dual-target multiplex assay. Scatter plot illustrating the linear relationship between the quantification cycles (Cq) of Set 1 (ROX) and Set 2 (HEX) across 20 confirmed Peruvian clinical isolates (n = 10 per subclade). The solid black line represents the linear regression fit (Pearson’s r = 0.70, *P* < 0.001).

In a multiplex format, the optimized primers and probe sets were evaluated to ensure that the simultaneous detection of both HA targets did not introduce competitive inhibition. An analysis of scatter plots revealed a positive linear correlation between Set 1 (ROX) and Set 2 (HEX) Cq values across the entire cohort (Pearson’s r = 0.70; R^²^ = 0.49; *P* < 0.001) (Figure 3). The linear fit statistics (y = 0.85 × + 3.16) indicated high analytical concordance, confirming that both targets amplified their respective regions efficiently within a single reaction tube.

### Assay performance across A(H3N2) subclades

**Figure 4:**
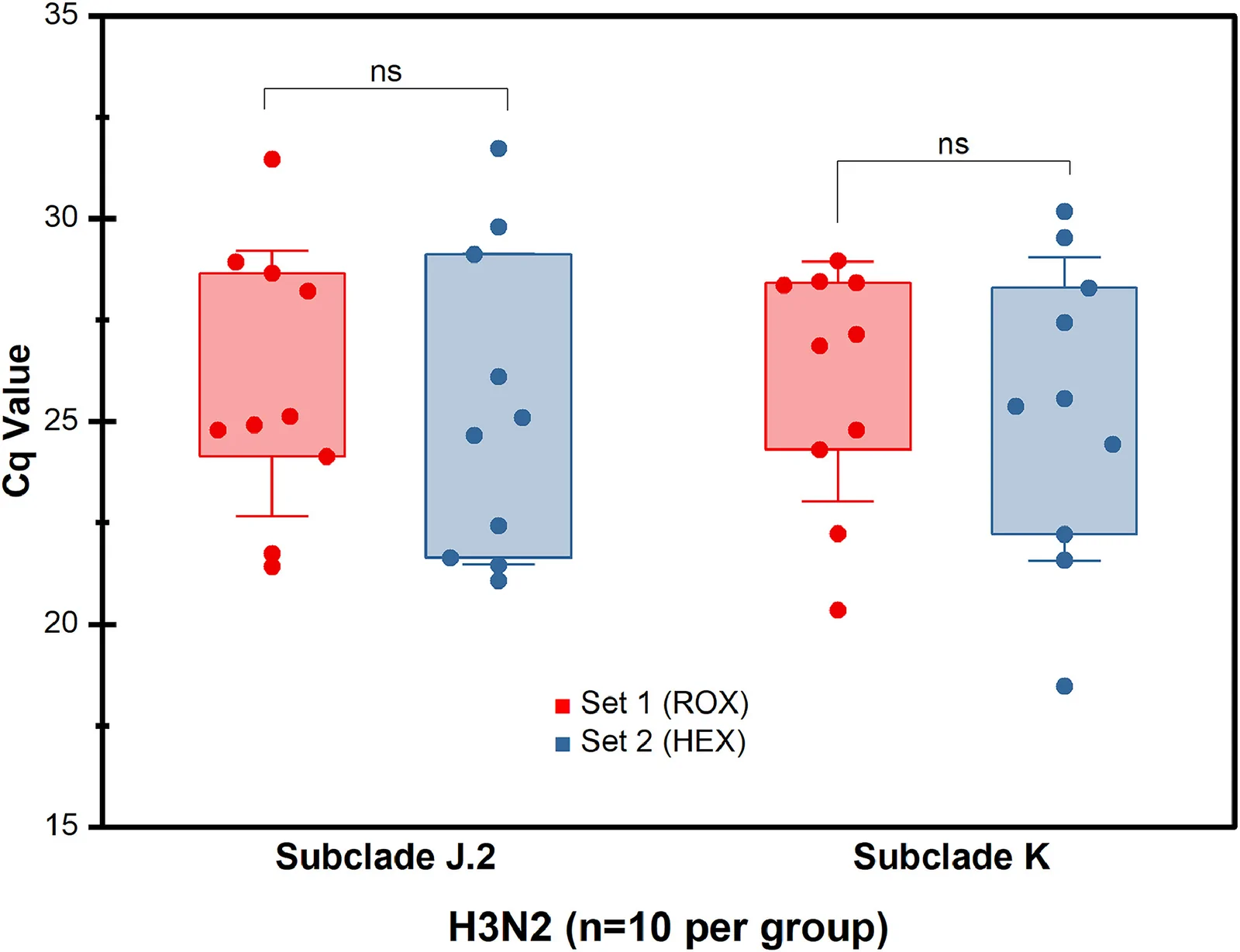
Multiplex assay performance across A(H3N2) subclades. Grouped box plots displaying the distribution of quantification cycles (Cq) values for the modified Set 1 (ROX) and Set 2 (HEX) targets when tested against the J.2 and K subclades (n = 10 isolates per group). A paired-sample t-test was used to compare the detection efficiency between targets within each subclade. ns, not significant (*P* > 0.05).

The Cq distributions of modified Set 1 (ROX) and modified Set 2 (HEX) within each A(H3N2) subclade were compared to determine which target showed preferential amplification (Figure 4). Both modified sets had equivalent detection efficiencies based on a grouped box plot analysis. There were no significant differences between the two optimized targets for subclade J.2 (paired Student’s *t*-test, *P* = 0.485). Subclade K showed similar results (paired Student’s *t*-test, *P* = 0.621).

Based on these results, both optimized targets performed comparably in the J.2 and K specimens, confirming that the dual-target format successfully provides technical redundancy across two distinct HA regions.

## Discussion

The emergence of influenza A(H3N2) subclades J.2 and K has critically compromised the diagnostic utility of standard molecular protocols. Our empirical evaluation demonstrated a complete failure of the WHO-recommended (2024) oligonucleotides against circulating Peruvian influenza viruses. By implementing targeted bioinformatic adjustments derived from genomic sequencing, an optimized dual-target multiplex RT-qPCR assay restored diagnostic sensitivity for frontline surveillance.

In diagnostic workflows, continuous viral evolution creates a recurrent vulnerability to molecular target dropouts. Similar primer mismatches occurred during the early circulation period of the severe acute respiratory syndrome coronavirus 2 Delta variants, resulting in critical gaps in surveillance [11,12]. For influenza A(H3N2), persistent genetic drift within the HA gene erodes primer and probe binding sites, reducing the efficacy of established assays [8,19]. Recently, due to the rapid global expansion of the K subclade, prolonged, high-burden influenza seasons have occurred in the Southern Hemisphere [7]. This underscores the urgency of updating regional diagnostic frameworks to prevent false-negative reporting and delayed clinical interventions.

The routine integration of third-generation sequencing technologies is essential for identifying the precise genomic alterations responsible for these diagnostic failures. The ONT sequencing technology provides the necessary resolution to rapidly characterize low-complexity viral regions and confirm the specific point mutations disrupting hybridization kinetics [9,16,17]. It should be noted that in our cohort, WGS was used solely for pathogen identification and mutational profiling, which directly guided the targeted bioinformatic modifications. The Set 1 forward primer was modified by adding a 5′ G and deleting a 3′ AA to correct binding site erosion. Furthermore, three nucleotide substitutions were incorporated into both the Set 1 (A→G, T→A, A→G) and Set 2 (T→C, A→G, C→T) probes to perfectly match the contemporary J.2 and K subclades. As a result, optimal hybridization kinetics and thermodynamic stability were restored without the need to design entirely new target regions.

Diagnostic assays based on single-target amplification are highly susceptible to future mutations. The dual-target multiplex strategy evaluated here mitigates this risk by simultaneously amplifying two distinct, non-competing regions of the HA gene. With this redesign, we achieved high analytical concordance and equivalent clinical performance for both the J.2 and the highly mutated K subclades. Therefore, a spontaneous structural variation compromising one oligonucleotide set is compensated by the complementary sequence. This redundancy establishes a resilient diagnostic mechanism required for reliable routine screening [15,20], but it does not eliminate the need for periodic sequence-based assay review.

The continued circulation of genetically divergent A(H3N2) strains, alongside the persistent risk of zoonotic spillovers, highlights the need for robust molecular tools under a One Health framework [13,21]. The rapid global expansion of subclade K, which recently contributed to extended influenza seasons in Australia and New Zealand [7], illustrates the capacity of these viruses to generate epidemiologically relevant variants within short timeframes. Genomic surveillance across diverse geographical settings confirms that these contemporary lineages diverge rapidly from vaccine strains and established diagnostic targets [5,[8], [9], [10]]. Therefore, maintaining diagnostic integrity requires dynamic, iterative revisions to PCR protocols rooted in continuous local genomic monitoring.

### Limitations

The sample size of the clinical validation panel is constrained, and the assay’s performance was evaluated exclusively against J.2 and K isolates. Further multicenter validation incorporating a broader diversity of global A(H3N2) lineages is required. This study evaluated only molecular detection performance, not clinical outcomes, viral transmissibility, or vaccine effectiveness. These results demonstrate diagnostic assay recovery within the evaluated genomic context, not altered clinical severity or immune escape.

## Conclusions

Contemporary influenza A(H3N2) HA sequence genetic drift severely compromises molecular subtyping when primer and probe binding region sequences are not updated. In this study, J.2- and K-subclade-related samples exhibited complete target dropout using the standard WHO (2024) oligonucleotides. However, a redesigned dual-target multiplex RT-qPCR assay fully restored amplification in all sequencing-confirmed A(H3N2) isolates. Routine genomic surveillance is necessary to continuously audit binding sites and update assays in a timely manner. Consequently, epidemiological surveillance and clinical management can be maintained in response to the accelerated molecular evolution of the influenza viruses, especially in settings where RT-qPCR remains the primary tool for frontline influenza surveillance.

## CRediT authorship contribution statement

**Miguel Angel Aguilar-Luis:** Writing – review & editing, Writing – original draft, Visualization, Project administration, Investigation, Formal analysis, Data curation, Conceptualization. **Juana del Valle-Mendoza:** Writing – review & editing, Writing – original draft, Validation, Supervision, Project administration, Investigation, Funding acquisition. **Yordi Tarazona-Castro:** Writing – original draft, Methodology, Formal analysis. **Deysi Aguilar-Luis:** Writing – original draft, Methodology, Formal analysis. **Eliezer Bonifacio-Velez de Villa:** Writing – original draft, Methodology. **Angela Cornejo-Tapia:** Writing – original draft, Methodology, Investigation. **Andrés Antón:** Writing – review & editing. **Cristina Andrés:** Writing – review & editing, Validation. **Alberto Laguna-Torres:** Writing – original draft, Resources, Funding acquisition.

## Declaration of competing interest

The authors have no competing interests to declare.

## Appendix Supplementary materials

Image, application 1
