# Supplementary material for: Update and optimization of a multiplex RT-qPCR assay to overcome diagnostic failure in emerging influenza A(H3N2) subclades J.2 and K (Peru, 2024–2026)
Source: IJID Reg. 2026 Sep 1;21:100993. doi: 10.1016/j.ijregi.2026.100993 (PMC13594907; doi:10.1016/j.ijregi.2026.100993)
Supplement: Supplementary file 1 [file mmc1.docx]

**Table S1.** The initial RT-qPCR quantification cycle (Cq) values for influenza A(H3N2) positive clinical specimens.

| **N°** | **Sample ID** | **Specimen**  **collection date** | **Target gene / Assay** | **Cq Value** | **Included in**  **validation**  **panel** |
| --- | --- | --- | --- | --- | --- |
| 1 | GI-2585 | 19-May-25 | Matrix / InfA | 25.5 | Yes |
| 2 | GI-2650 | 17-Jun-25 | Matrix / InfA | 26.6 | No |
| 3 | GI-2661 | 17-Jun-25 | Matrix / InfA | 29.35 | No |
| 4 | GI-2724 | 21-Jul-25 | Matrix / InfA | 22.17 | No |
| 5 | GI-2768 | 7-Aug-25 | Matrix / InfA | 23.18 | Yes |
| 6 | GI-2752 | 7-Aug-25 | Matrix / InfA | 21.71 | No |
| 7 | GI-2791 | 28-Aug-25 | Matrix / InfA | 21.32 | Yes |
| 8 | GI-2825 | 23-Sep-25 | Matrix / InfA | 27.6 | Yes |
| 9 | GI-2884 | 4-Nov-25 | Matrix / InfA | 26.81 | Yes |
| 10 | GI-2919 | 15-Dec-25 | Matrix / InfA | 26.65 | Yes |
| 11 | GI-2940 | 15-Dec-25 | Matrix / InfA | 20.27 | Yes |
| 12 | GI-2945 | 17-Dec-25 | Matrix / InfA | 32.53 | Yes |
| 13 | GI-2956 | 6-Jan-26 | Matrix / InfA | 22.73 | Yes |
| 14 | GI-2973 | 6-Jan-26 | Matrix / InfA | 31.97 | Yes |
| 15 | GI-2957 | 6-Jan-26 | Matrix / InfA | 28.66 | Yes |
| 16 | GI-3018 | 17-Feb-26 | Matrix / InfA | 25.85 | Yes |
| 17 | GI-3020 | 17-Feb-26 | Matrix / InfA | 21.27 | Yes |
| 18 | GI-3034 | 17-Feb-26 | Matrix / InfA | 38.71 | No |
| 19 | GI-3037 | 24-Feb-26 | Matrix / InfA | 31.38 | Yes |
| 20 | GI-3039 | 24-Feb-26 | Matrix / InfA | 30.21 | Yes |
| 21 | GI-3048 | 24-Feb-26 | Matrix / InfA | 31.5 | Yes |
| 22 | GI-3050 | 24-Feb-26 | Matrix / InfA | 34.43 | No |
| 23 | GI-3054 | 27-Feb-26 | Matrix / InfA | 28.35 | Yes |
| 24 | GI-3071 | 6-Mar-26 | Matrix / InfA | 32.86 | No |
| 25 | GI-3062 | 6-Mar-26 | Matrix / InfA | 27.3 | No |
| 26 | GI-3087 | 18-Mar-26 | Matrix / InfA | 32.12 | Yes |
| 27 | GI-3096 | 18-Mar-26 | Matrix / InfA | 27.66 | Yes |
| 28 | GI-3098 | 18-Mar-26 | Matrix / InfA | 24.68 | Yes |

Note: Cq: Quantification cycle. Initial screening was performed using routine diagnostic primers targeting conserved genomic regions. Although whole-genome sequencing prioritized specimens with Cq < 30, complete genomes were recovered for all 28 cases.

Table S2. Sequencing quality control metrics and GISAID accession numbers for the 20 whole-genome sequenced influenza A(H3N2) clinical isolates.

| Sample ID | Subclade | GISAID  accession No. | Mean  sequencing  depth (X) | Target coverage (HA) | Mean read  Quality | Total Yield |
| --- | --- | --- | --- | --- | --- | --- |
| GI-2585 | J.2 | EPI_ISL_20144594 | 306 | 100.0% (1-1701/1701) | 23.4 | 0.031 Gb |
| GI-2768 | J.2 | EPI_ISL_20147930 | 1135 | 100.0% (1-1701/1701) | 22.4 | 0.035 Gb |
| GI-2791 | J.2 | EPI_ISL_20286606 | 710 | 100.0% (1-1701/1701) | 21.6 | 0.028 Gb |
| GI-2825 | J.2 | EPI_ISL_20286608 | 335 | 100.0% (1-1701/1701) | 22.0 | 0.0423 Gb |
| GI-2884 | J.2 | EPI_ISL_20286609 | 427 | 100.0% (1-1701/1701) | 21.6 | 0.026 Gb |
| GI-2919 | K | EPI_ISL_20339289 | 18053 | 100.0% (1-1701/1701) | 21.4 | 0.0575 Gb |
| GI-2940 | K | EPI_ISL_20339290 | 9485 | 100.0% (1-1701/1701) | 23.8 | 0.0612 Gb |
| GI-2945 | K | EPI_ISL_20339291 | 696 | 100.0% (1-1701/1701) | 23.1 | 0.108 Gb |
| GI-2956 | J.2 | EPI_ISL_20339303 | 14616 | 100.0% (1-1701/1701) | 22.8 | 0.089 Gb |
| GI-2957 | K | EPI_ISL_20339345 | 19227 | 100.0% (1-1701/1701) | 23.6 | 0.129 Gb |
| GI-2973 | J.2 | EPI_ISL_20339384 | 1963 | 100.0% (1-1701/1701) | 24.0 | 0.0267 Gb |
| GI-3018 | K | EPI_ISL_20458483 | 4806 | 100.0% (1-1701/1701) | 22.2 | 0.0784 Gb |
| GI-3020 | K | EPI_ISL_20458508 | 1150 | 100.0% (1-1701/1701) | 21.8 | 0.078 Gb |
| GI-3037 | K | EPI_ISL_20458510 | 3888 | 100.0% (1-1701/1701) | 21.8 | 0.0583 Gb |
| GI-3039 | K | EPI_ISL_20458511 | 35 | 100.0% (1-1701/1701) | 22.1 | 0.0289 Gb |
| GI-3048 | K | EPI_ISL_20458512 | 735 | 100.0% (1-1701/1701) | 21.9 | 0.0173 Gb |
| GI-3054 | K | EPI_ISL_20458514 | 288 | 100.0% (1-1701/1701) | 21.8 | 0.0399 Gb |
| GI-3087 | J.2 | EPI_ISL_20458518 | 215 | 100.0% (1-1701/1701) | 22.1 | 0.0438 Gb |
| GI-3096 | J.2.4 | EPI_ISL_20458519 | 1481 | 100.0% (1-1701/1701) | 21.4 | 0.0197 Gb |
| GI-3098 | J.2 | EPI_ISL_20458520 | 2117 | 100.0% (1-1701/1701) | 20.8 | 0.0454 Gb |

Note: Total yield represents the total base yield generated for each barcoded library. HA target coverage indicates the percentage of the hemagglutinin reference segment successfully assembled. Basecalling was executed using the super-accurate (SUP) model.

**Supplementary Figure S1. *In silico* sequence alignment of the Set 1 oligonucleotide binding regions against global influenza A(H3N2) isolates.** The alignment demonstrates high sequence conservation and exact complementarity of the optimized H3-266-F, H3-315-Probe-Mod, and H3-373-R oligonucleotides across representative J.2 and K subclade genomes circulating globally (2025–2026).

**Supplementary Figure S2. *In silico* sequence alignment of the Set 2 oligonucleotide binding regions against global influenza A(H3N2) isolates.** The alignment confirms the absence of critical mismatches and complete inclusivity of the optimized H3-666-F, H3-732-Probe-Mod, and H3-911-R oligonucleotides across diverse international J.2 and K subclade isolates (2025–2026).
